# {1-[(3,5-Dimethyl-4*H*-1,2,4-triazol-4-yl)imino]eth­yl}ferrocene

**DOI:** 10.1107/S1600536808028973

**Published:** 2008-09-17

**Authors:** Xin-Qi Hao, Dong-Song Liang, Ruo-Yi Liu, Jun-Fang Gong, Mao-Ping Song

**Affiliations:** a Department of Chemistry, Henan Key Laboratory of Chemical Biology and Organic Chemistry, Zhengzhou University, Zhengzhou 450052, People’s Republic of China; b College of Materials Science and Engineering, Zhengzhou University, Zhengzhou 450052, People’s Republic of China

## Abstract

In the title compound, [Fe(C_5_H_5_)(C_11_H_13_N_4_)], the triazolyl and Cp ring form a dihedral angle of 76.6 (3)°. In the crystal structure, there are both intra- and inter­molecular C—H⋯π inter­actions, forming a one-dimensional chain structure along [010].

## Related literature

For related literature, see: Hao *et al.* (2007 ▶); Huo *et al.* (1994 ▶); Wu *et al.* (2001 ▶).
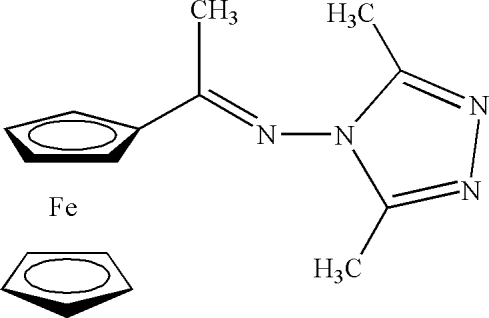


## Experimental

### 

#### Crystal data

[Fe(C_5_H_5_)(C_11_H_13_N_4_)]
*M* *_r_* = 322.19
Monoclinic, 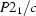

*a* = 8.7851 (18) Å
*b* = 13.271 (3) Å
*c* = 13.035 (3) Å
β = 104.49 (3)°
*V* = 1471.4 (5) Å^3^
*Z* = 4
Mo *K*α radiation
μ = 1.02 mm^−1^
*T* = 293 (2) K
0.30 × 0.26 × 0.20 mm

#### Data collection

Rigaku Saturn724 CCD area-detector diffractometer
Absorption correction: multi-scan (*SADABS*; Sheldrick, 1996 ▶) *T*_min_ = 0.749, *T*_max_ = 0.822
14449 measured reflections
2583 independent reflections
2485 reflections with *I* > 2σ(*I*)
*R*_int_ = 0.026

#### Refinement

*R*[*F* ^2^ > 2σ(*F* ^2^)] = 0.029
*wR*(*F* ^2^) = 0.068
*S* = 1.09
2583 reflections
193 parameters
H-atom parameters constrained
Δρ_max_ = 0.22 e Å^−3^
Δρ_min_ = −0.18 e Å^−3^

### 

Data collection: *CrystalClear* (Rigaku/MSC, 2006 ▶); cell refinement: *CrystalClear*; data reduction: *CrystalClear*; program(s) used to solve structure: *SHELXS97* (Sheldrick, 2008 ▶); program(s) used to refine structure: *SHELXL97* (Sheldrick, 2008 ▶); molecular graphics: Bruker *SHELXTL* (Sheldrick, 2008 ▶); software used to prepare material for publication: *SHELXTL*.

## Supplementary Material

Crystal structure: contains datablocks global, I. DOI: 10.1107/S1600536808028973/si2106sup1.cif

Structure factors: contains datablocks I. DOI: 10.1107/S1600536808028973/si2106Isup2.hkl

Additional supplementary materials:  crystallographic information; 3D view; checkCIF report

## Figures and Tables

**Table 1:** Hydrogen-bond geometry (Å, °)

*D*—H⋯*A*	*D*—H	H⋯*A*	*D*⋯*A*	*D*—H⋯*A*
C6—H6⋯*Cg*1	0.98	2.52	3.255 (3)	132
C5—H5⋯*Cg*3^i^	0.98	2.87	3.703 (5)	144

Symmetry code: (i) 
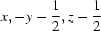
. *Cg*1 and *Cg*3 are the centroids of the triazolyl ring and the Cp rings C6–C10, respectively.

**Fig. 1.**
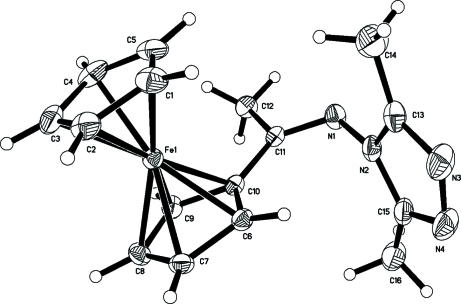
The molecular structure of the title compound with displacement ellipsoids at the 30% probability level.

**Fig. 2.**
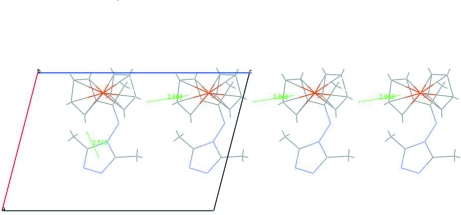
Partial view of the crystal packing showing the formation of the 1D chain structure formed by the C—H···π interactions.

**Fig. 3.**
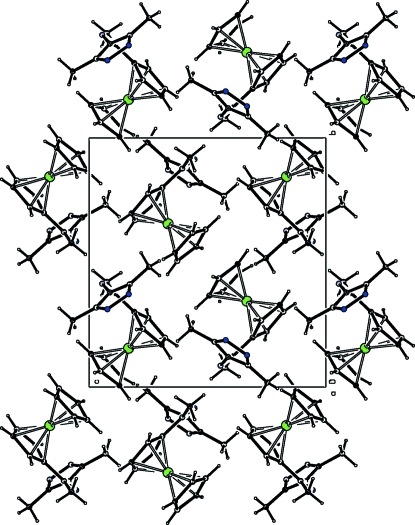
Section of the crystal packing viewed down [100].

## supplementary crystallographic information

### Comment

During the past decades, we have systematically studied the cyclometallation reaction of Schiff base type of ferrocenylimines (Huo *et al.*, 1994; Wu *et al.*, 2001). Following these work, here we report the synthesis, characterization and crystal structure of the title ferrocenylimine ligand.

A view of the molecular structure of the title compound is given in Fig. 1. All the bond distances and angles are within normal ranges, the C11—N1 distance [1.290 (2) Å] is similar to those of the related complex (Hao *et al.*, 2007) and the C11—N1—N2 angle is 115.15 (16)°. The triazolyl and Cp ring form a dihedral angle of 103.4 (3)°. Fig. 2 and Table 1 shows that in the crystal there exist intra- and intermolecular C—H···π interactions: H6···*Cg*1 = 2.519 Å [symmetry code for *Cg*1 (*x*, *y*, *z*), intra], and H5···*Cg*3 = 2.868 Å; symmetry code for *Cg*3 (*x*, -1/2 - *y*, -1/2 + *z*)]. *Cg*1 and *Cg*3 are the centroids of the triazolyl and one of the Cp rings C6–C10, respectively], which are attributed to construct the one-dimensional chain structure of the title compound. Partial stacking between neighbouring ferrocene units *via* inversion centres is shown in Fig. 3. The partial π–π stacking interactions are found between *Cg*2 and *Cg*2^i^ [symmetry code i = (-*x*, -*y*, 2 - *z*)], with a distance 3.8525 (18) Å, the perpendicular distance is 3.295 Å with a slippage of 1.829 Å. *Cg*2 is the centroid of the Cp ring C1–C5.

### Experimental

Acetylferrocene (1 mmol) was dissolved in anhydrous toluene (40 ml) and 3,5-dimethyl-4-amino-4*H*-1,2,4-triazole (1.5 mmol) was added. The red solution was refluxed under argon atmosphere for about 3 days. Every day an addition of small quantities of activated Al_2_O_3_ was necessary to complete the reaction. The hot solution was carefully filtered, and the filtrate was concentrated to dryness in a rotary evaporator. The residue was purified by passing through a silica gel column with CH_2_Cl_2_ as eluent, giving the title compound, which was recrystallized from dichloromethane–petroleum ether solution at room temperature to give the desired product as colorless crystals suitable for single-crystal X-ray diffraction (yield 25%; m.p: 440 K-443 K). IR data (v_max/ cm^-1^): 1591, 1569, 1531, 1479, 1409, 1307, 1104, 1005, 827, 762. NMR δ(H) 4.87 (2*H*, s), 4.59 (2*H*, s), 4.25 (5*H*, s), 2.35 (6*H*, s), 2.07 (3*H*, s). MS-ESI^+^ [m/*z*]: 323 (*M*+H), 345 (*M*+Na), 667 (2*M*+Na).

### Refinement

H atoms attached to C atoms of the title compound were placed in geometrically idealized positions and treated as riding with C—H distances constrained to 0.93–0.96 Å, and with *U*_iso_(H) = 1.2Ueq(C) (1.5Ueq for methyl H).

### Crystal data

**Table:**

[Fe(C_5_H_5_)(C_11_H_13_N_4_)]	*F*(000) = 672
*M**_r_* = 322.19	*D*_x_ = 1.454 Mg m^−^^3^
Monoclinic, *P*2_1_/*c*	Mo *K*α radiation, λ = 0.71073 Å
*a* = 8.7851 (18) Å	Cell parameters from 4008 reflections
*b* = 13.271 (3) Å	θ = 2.5–26.1°
*c* = 13.035 (3) Å	µ = 1.02 mm^−^^1^
β = 104.49 (3)°	*T* = 293 K
*V* = 1471.4 (5) Å^3^	Block, red
*Z* = 4	0.30 × 0.26 × 0.20 mm

### Data collection

**Table:**

Rigaku/MSC MODEL? CCD area-detector diffractometer	2583 independent reflections
Radiation source: fine-focus sealed tube	2485 reflections with *I* > 2σ(*I*)
graphite	*R*_int_ = 0.026
φ and ω scans	θ_max_ = 25.0°, θ_min_ = 3.0°
Absorption correction: multi-scan (SADABS; Sheldrick, 1996)	*h* = −10→10
*T*_min_ = 0.749, *T*_max_ = 0.822	*k* = −15→15
14449 measured reflections	*l* = −15→15

### Refinement

**Table:**

Refinement on *F*^2^	Primary atom site location: structure-invariant direct methods
Least-squares matrix: full	Secondary atom site location: difference Fourier map
*R*[*F*^2^ > 2σ(*F*^2^)] = 0.029	Hydrogen site location: inferred from neighbouring sites
*wR*(*F*^2^) = 0.068	H-atom parameters constrained
*S* = 1.09	*w* = 1/[σ^2^(*F*_o_^2^) + (0.031*P*)^2^ + 0.6853*P*] where *P* = (*F*_o_^2^ + 2*F*_c_^2^)/3
2583 reflections	(Δ/σ)_max_ = 0.001
193 parameters	Δρ_max_ = 0.22 e Å^−^^3^
0 restraints	Δρ_min_ = −0.18 e Å^−^^3^

### Special details

**Table:**

Geometry. All e.s.d.'s (except the e.s.d. in the dihedral angle between two l.s. planes) are estimated using the full covariance matrix. The cell e.s.d.'s are taken into account individually in the estimation of e.s.d.'s in distances, angles and torsion angles; correlations between e.s.d.'s in cell parameters are only used when they are defined by crystal symmetry. An approximate (isotropic) treatment of cell e.s.d.'s is used for estimating e.s.d.'s involving l.s. planes.
Refinement. Refinement of *F*^2^ against ALL reflections. The weighted *R*-factor *wR* and goodness of fit *S* are based on *F*^2^, conventional *R*-factors *R* are based on *F*, with *F* set to zero for negative *F*^2^. The threshold expression of *F*^2^ > σ(*F*^2^) is used only for calculating *R*-factors(gt) *etc*. and is not relevant to the choice of reflections for refinement. *R*-factors based on *F*^2^ are statistically about twice as large as those based on *F*, and *R*- factors based on ALL data will be even larger.

### Fractional atomic coordinates and isotropic or equivalent isotropic displacement parameters (Å^2^)

**Table:**

	*x*	*y*	*z*	*U*_iso_*/*U*_eq_	
Fe1	0.14717 (3)	0.155436 (19)	0.83336 (2)	0.02657 (10)	
N1	0.36589 (18)	0.41476 (13)	0.94499 (13)	0.0366 (4)	
N2	0.49929 (18)	0.37952 (13)	0.91279 (13)	0.0351 (4)	
N3	0.7280 (2)	0.31235 (17)	0.92302 (18)	0.0566 (5)	
N4	0.6890 (2)	0.37255 (17)	0.83286 (17)	0.0538 (5)	
C1	0.2665 (3)	0.06374 (16)	0.95248 (17)	0.0456 (5)	
H1	0.3811	0.0571	0.9763	0.055*	
C2	0.1664 (3)	0.00600 (16)	0.87231 (18)	0.0454 (5)	
H2	0.1988	−0.0480	0.8310	0.054*	
C3	0.0112 (3)	0.03956 (17)	0.86285 (19)	0.0482 (6)	
H3	−0.0834	0.0131	0.8135	0.058*	
C4	0.0157 (3)	0.11866 (18)	0.93709 (19)	0.0498 (6)	
H4	−0.0749	0.1562	0.9482	0.060*	
C5	0.1738 (3)	0.13290 (17)	0.99218 (17)	0.0469 (6)	
H5	0.2127	0.1826	1.0482	0.056*	
C6	0.3048 (2)	0.23320 (14)	0.77318 (15)	0.0302 (4)	
H6	0.4194	0.2305	0.7996	0.036*	
C7	0.2125 (2)	0.17029 (15)	0.69381 (15)	0.0346 (4)	
H7	0.2523	0.1162	0.6564	0.042*	
C8	0.0526 (2)	0.19765 (16)	0.67915 (15)	0.0360 (5)	
H8	−0.0371	0.1655	0.6300	0.043*	
C9	0.0439 (2)	0.27709 (14)	0.74964 (15)	0.0324 (4)	
H9	−0.0528	0.3100	0.7568	0.039*	
C10	0.2013 (2)	0.30161 (13)	0.80928 (14)	0.0260 (4)	
C11	0.2331 (2)	0.37758 (14)	0.89329 (14)	0.0270 (4)	
C12	0.0945 (2)	0.42209 (15)	0.92519 (16)	0.0359 (4)	
H12A	0.1309	0.4672	0.9838	0.054*	
H12B	0.0338	0.3691	0.9457	0.054*	
H12C	0.0301	0.4585	0.8665	0.054*	
C13	0.6131 (2)	0.31904 (17)	0.97039 (19)	0.0440 (5)	
C14	0.6040 (3)	0.2679 (2)	1.0694 (2)	0.0640 (7)	
H14A	0.6814	0.2153	1.0854	0.096*	
H14B	0.5011	0.2394	1.0608	0.096*	
H14C	0.6236	0.3158	1.1264	0.096*	
C15	0.5514 (2)	0.41269 (16)	0.82868 (17)	0.0402 (5)	
C16	0.4626 (3)	0.48245 (19)	0.7469 (2)	0.0550 (6)	
H16A	0.5297	0.5061	0.7042	0.083*	
H16B	0.4264	0.5387	0.7806	0.083*	
H16C	0.3740	0.4478	0.7030	0.083*	

### Atomic displacement parameters (Å^2^)

**Table:**

	*U*^11^	*U*^22^	*U*^33^	*U*^12^	*U*^13^	*U*^23^
Fe1	0.02826 (16)	0.02453 (16)	0.02769 (16)	−0.00021 (11)	0.00847 (11)	0.00028 (11)
N1	0.0289 (8)	0.0381 (10)	0.0449 (10)	−0.0003 (7)	0.0130 (7)	−0.0112 (8)
N2	0.0232 (8)	0.0385 (9)	0.0442 (10)	−0.0042 (7)	0.0098 (7)	−0.0095 (8)
N3	0.0298 (10)	0.0669 (13)	0.0758 (15)	0.0070 (9)	0.0181 (10)	0.0068 (12)
N4	0.0339 (10)	0.0674 (14)	0.0661 (14)	−0.0006 (9)	0.0237 (9)	0.0013 (11)
C1	0.0567 (14)	0.0391 (12)	0.0384 (12)	0.0102 (10)	0.0072 (10)	0.0107 (10)
C2	0.0691 (15)	0.0249 (10)	0.0455 (12)	0.0014 (10)	0.0208 (11)	0.0024 (9)
C3	0.0534 (14)	0.0409 (12)	0.0550 (14)	−0.0139 (11)	0.0223 (11)	0.0045 (11)
C4	0.0596 (15)	0.0439 (13)	0.0588 (15)	0.0053 (11)	0.0393 (13)	0.0130 (12)
C5	0.0758 (17)	0.0389 (12)	0.0298 (11)	0.0017 (11)	0.0202 (11)	0.0021 (9)
C6	0.0281 (9)	0.0323 (10)	0.0325 (10)	−0.0012 (8)	0.0116 (8)	−0.0007 (8)
C7	0.0439 (11)	0.0349 (11)	0.0274 (10)	−0.0011 (9)	0.0136 (9)	−0.0021 (8)
C8	0.0378 (11)	0.0371 (11)	0.0284 (10)	−0.0042 (9)	−0.0006 (8)	0.0025 (9)
C9	0.0277 (9)	0.0308 (10)	0.0362 (10)	0.0014 (8)	0.0033 (8)	0.0055 (8)
C10	0.0249 (9)	0.0247 (9)	0.0295 (9)	0.0008 (7)	0.0088 (7)	0.0040 (8)
C11	0.0262 (9)	0.0251 (9)	0.0315 (10)	0.0002 (7)	0.0104 (8)	0.0034 (8)
C12	0.0314 (10)	0.0386 (11)	0.0397 (11)	0.0054 (9)	0.0125 (9)	−0.0026 (9)
C13	0.0271 (10)	0.0469 (13)	0.0550 (14)	−0.0032 (9)	0.0050 (10)	−0.0010 (11)
C14	0.0489 (14)	0.0784 (19)	0.0629 (16)	0.0037 (13)	0.0106 (12)	0.0135 (15)
C15	0.0291 (10)	0.0438 (12)	0.0490 (12)	−0.0102 (9)	0.0122 (9)	−0.0073 (10)
C16	0.0437 (13)	0.0599 (15)	0.0623 (15)	−0.0071 (11)	0.0150 (12)	0.0090 (13)

### Geometric parameters (Å, °)

**Table:**

Fe1—C9	2.0310 (19)	C4—H4	0.9800
Fe1—C6	2.0350 (19)	C5—H5	0.9800
Fe1—C10	2.0402 (18)	C6—C7	1.416 (3)
Fe1—C3	2.042 (2)	C6—C10	1.444 (3)
Fe1—C2	2.044 (2)	C6—H6	0.9800
Fe1—C1	2.044 (2)	C7—C8	1.417 (3)
Fe1—C4	2.045 (2)	C7—H7	0.9800
Fe1—C5	2.046 (2)	C8—C9	1.413 (3)
Fe1—C7	2.0494 (19)	C8—H8	0.9800
Fe1—C8	2.051 (2)	C9—C10	1.444 (3)
N1—C11	1.290 (2)	C9—H9	0.9800
N1—N2	1.419 (2)	C10—C11	1.463 (3)
N2—C13	1.354 (3)	C11—C12	1.503 (2)
N2—C15	1.363 (3)	C12—H12A	0.9600
N3—C13	1.311 (3)	C12—H12B	0.9600
N3—N4	1.391 (3)	C12—H12C	0.9600
N4—C15	1.310 (3)	C13—C14	1.479 (3)
C1—C5	1.409 (3)	C14—H14A	0.9600
C1—C2	1.412 (3)	C14—H14B	0.9600
C1—H1	0.9800	C14—H14C	0.9600
C2—C3	1.410 (3)	C15—C16	1.478 (3)
C2—H2	0.9800	C16—H16A	0.9600
C3—C4	1.421 (3)	C16—H16B	0.9600
C3—H3	0.9800	C16—H16C	0.9600
C4—C5	1.407 (4)		
			
C9—Fe1—C6	69.23 (8)	C5—C4—C3	107.6 (2)
C9—Fe1—C10	41.54 (7)	C5—C4—Fe1	69.94 (12)
C6—Fe1—C10	41.52 (7)	C3—C4—Fe1	69.53 (12)
C9—Fe1—C3	119.75 (9)	C5—C4—H4	126.2
C6—Fe1—C3	160.23 (9)	C3—C4—H4	126.2
C10—Fe1—C3	156.19 (9)	Fe1—C4—H4	126.2
C9—Fe1—C2	154.55 (9)	C4—C5—C1	108.2 (2)
C6—Fe1—C2	124.47 (9)	C4—C5—Fe1	69.82 (13)
C10—Fe1—C2	162.36 (9)	C1—C5—Fe1	69.75 (12)
C3—Fe1—C2	40.38 (9)	C4—C5—H5	125.9
C9—Fe1—C1	163.11 (9)	C1—C5—H5	125.9
C6—Fe1—C1	109.00 (9)	Fe1—C5—H5	125.9
C10—Fe1—C1	126.01 (9)	C7—C6—C10	108.47 (16)
C3—Fe1—C1	67.77 (10)	C7—C6—Fe1	70.26 (11)
C2—Fe1—C1	40.41 (9)	C10—C6—Fe1	69.43 (10)
C9—Fe1—C4	107.15 (9)	C7—C6—H6	125.8
C6—Fe1—C4	158.00 (9)	C10—C6—H6	125.8
C10—Fe1—C4	121.37 (8)	Fe1—C6—H6	125.8
C3—Fe1—C4	40.71 (10)	C6—C7—C8	108.32 (17)
C2—Fe1—C4	68.24 (9)	C6—C7—Fe1	69.17 (11)
C1—Fe1—C4	67.84 (10)	C8—C7—Fe1	69.87 (11)
C9—Fe1—C5	125.62 (9)	C6—C7—H7	125.8
C6—Fe1—C5	123.19 (9)	C8—C7—H7	125.8
C10—Fe1—C5	108.82 (8)	Fe1—C7—H7	125.8
C3—Fe1—C5	67.89 (10)	C9—C8—C7	108.47 (17)
C2—Fe1—C5	68.00 (9)	C9—C8—Fe1	68.98 (11)
C1—Fe1—C5	40.30 (9)	C7—C8—Fe1	69.71 (11)
C4—Fe1—C5	40.24 (10)	C9—C8—H8	125.8
C9—Fe1—C7	68.49 (8)	C7—C8—H8	125.8
C6—Fe1—C7	40.57 (8)	Fe1—C8—H8	125.8
C10—Fe1—C7	69.16 (8)	C8—C9—C10	108.53 (17)
C3—Fe1—C7	123.13 (9)	C8—C9—Fe1	70.53 (11)
C2—Fe1—C7	106.84 (8)	C10—C9—Fe1	69.57 (10)
C1—Fe1—C7	121.81 (9)	C8—C9—H9	125.7
C4—Fe1—C7	160.03 (10)	C10—C9—H9	125.7
C5—Fe1—C7	157.74 (10)	Fe1—C9—H9	125.7
C9—Fe1—C8	40.50 (8)	C9—C10—C6	106.20 (16)
C6—Fe1—C8	68.38 (8)	C9—C10—C11	122.42 (16)
C10—Fe1—C8	69.05 (8)	C6—C10—C11	131.23 (16)
C3—Fe1—C8	106.12 (9)	C9—C10—Fe1	68.89 (10)
C2—Fe1—C8	119.77 (9)	C6—C10—Fe1	69.05 (10)
C1—Fe1—C8	155.79 (9)	C11—C10—Fe1	123.25 (13)
C4—Fe1—C8	123.78 (10)	N1—C11—C10	129.26 (17)
C5—Fe1—C8	161.24 (9)	N1—C11—C12	113.23 (16)
C7—Fe1—C8	40.43 (8)	C10—C11—C12	117.50 (16)
C11—N1—N2	115.15 (16)	C11—C12—H12A	109.5
C13—N2—C15	106.71 (17)	C11—C12—H12B	109.5
C13—N2—N1	125.56 (18)	H12A—C12—H12B	109.5
C15—N2—N1	126.86 (17)	C11—C12—H12C	109.5
C13—N3—N4	107.64 (18)	H12A—C12—H12C	109.5
C15—N4—N3	107.37 (18)	H12B—C12—H12C	109.5
C5—C1—C2	108.4 (2)	N3—C13—N2	109.1 (2)
C5—C1—Fe1	69.95 (12)	N3—C13—C14	126.6 (2)
C2—C1—Fe1	69.79 (12)	N2—C13—C14	124.2 (2)
C5—C1—H1	125.8	C13—C14—H14A	109.5
C2—C1—H1	125.8	C13—C14—H14B	109.5
Fe1—C1—H1	125.8	H14A—C14—H14B	109.5
C3—C2—C1	107.6 (2)	C13—C14—H14C	109.5
C3—C2—Fe1	69.74 (12)	H14A—C14—H14C	109.5
C1—C2—Fe1	69.80 (12)	H14B—C14—H14C	109.5
C3—C2—H2	126.2	N4—C15—N2	109.1 (2)
C1—C2—H2	126.2	N4—C15—C16	126.8 (2)
Fe1—C2—H2	126.2	N2—C15—C16	124.03 (19)
C2—C3—C4	108.2 (2)	C15—C16—H16A	109.5
C2—C3—Fe1	69.88 (12)	C15—C16—H16B	109.5
C4—C3—Fe1	69.76 (12)	H16A—C16—H16B	109.5
C2—C3—H3	125.9	C15—C16—H16C	109.5
C4—C3—H3	125.9	H16A—C16—H16C	109.5
Fe1—C3—H3	125.9	H16B—C16—H16C	109.5
			
C11—N1—N2—C13	−110.8 (2)	C8—Fe1—C6—C10	82.38 (12)
C11—N1—N2—C15	81.3 (2)	C10—C6—C7—C8	0.0 (2)
C13—N3—N4—C15	−0.5 (3)	Fe1—C6—C7—C8	59.08 (14)
C9—Fe1—C1—C5	38.3 (4)	C10—C6—C7—Fe1	−59.11 (13)
C6—Fe1—C1—C5	119.29 (15)	C9—Fe1—C7—C6	82.77 (12)
C10—Fe1—C1—C5	76.07 (17)	C10—Fe1—C7—C6	38.07 (11)
C3—Fe1—C1—C5	−81.56 (16)	C3—Fe1—C7—C6	−164.92 (12)
C2—Fe1—C1—C5	−119.4 (2)	C2—Fe1—C7—C6	−123.76 (12)
C4—Fe1—C1—C5	−37.43 (15)	C1—Fe1—C7—C6	−82.21 (14)
C7—Fe1—C1—C5	162.25 (14)	C4—Fe1—C7—C6	163.8 (2)
C8—Fe1—C1—C5	−161.34 (19)	C5—Fe1—C7—C6	−50.8 (3)
C9—Fe1—C1—C2	157.8 (3)	C8—Fe1—C7—C6	119.84 (17)
C6—Fe1—C1—C2	−121.27 (14)	C9—Fe1—C7—C8	−37.08 (12)
C10—Fe1—C1—C2	−164.49 (12)	C6—Fe1—C7—C8	−119.84 (17)
C3—Fe1—C1—C2	37.87 (14)	C10—Fe1—C7—C8	−81.77 (12)
C4—Fe1—C1—C2	82.01 (15)	C3—Fe1—C7—C8	75.24 (15)
C5—Fe1—C1—C2	119.4 (2)	C2—Fe1—C7—C8	116.40 (13)
C7—Fe1—C1—C2	−78.31 (16)	C1—Fe1—C7—C8	157.95 (12)
C8—Fe1—C1—C2	−41.9 (3)	C4—Fe1—C7—C8	43.9 (3)
C5—C1—C2—C3	−0.2 (2)	C5—Fe1—C7—C8	−170.7 (2)
Fe1—C1—C2—C3	−59.72 (15)	C6—C7—C8—C9	−0.4 (2)
C5—C1—C2—Fe1	59.54 (15)	Fe1—C7—C8—C9	58.21 (14)
C9—Fe1—C2—C3	−46.5 (3)	C6—C7—C8—Fe1	−58.65 (13)
C6—Fe1—C2—C3	−162.68 (13)	C6—Fe1—C8—C9	−82.91 (12)
C10—Fe1—C2—C3	164.2 (2)	C10—Fe1—C8—C9	−38.20 (11)
C1—Fe1—C2—C3	118.7 (2)	C3—Fe1—C8—C9	117.18 (13)
C4—Fe1—C2—C3	37.77 (15)	C2—Fe1—C8—C9	158.75 (12)
C5—Fe1—C2—C3	81.29 (16)	C1—Fe1—C8—C9	−171.3 (2)
C7—Fe1—C2—C3	−121.70 (14)	C4—Fe1—C8—C9	76.29 (15)
C8—Fe1—C2—C3	−79.70 (16)	C5—Fe1—C8—C9	48.7 (3)
C9—Fe1—C2—C1	−165.18 (18)	C7—Fe1—C8—C9	−120.27 (17)
C6—Fe1—C2—C1	78.62 (16)	C9—Fe1—C8—C7	120.27 (17)
C10—Fe1—C2—C1	45.5 (3)	C6—Fe1—C8—C7	37.36 (11)
C3—Fe1—C2—C1	−118.7 (2)	C10—Fe1—C8—C7	82.06 (12)
C4—Fe1—C2—C1	−80.92 (15)	C3—Fe1—C8—C7	−122.55 (13)
C5—Fe1—C2—C1	−37.41 (14)	C2—Fe1—C8—C7	−80.99 (14)
C7—Fe1—C2—C1	119.60 (14)	C1—Fe1—C8—C7	−51.1 (3)
C8—Fe1—C2—C1	161.61 (13)	C4—Fe1—C8—C7	−163.44 (12)
C1—C2—C3—C4	0.3 (2)	C5—Fe1—C8—C7	169.0 (2)
Fe1—C2—C3—C4	−59.46 (15)	C7—C8—C9—C10	0.7 (2)
C1—C2—C3—Fe1	59.75 (15)	Fe1—C8—C9—C10	59.40 (13)
C9—Fe1—C3—C2	158.96 (13)	C7—C8—C9—Fe1	−58.66 (14)
C6—Fe1—C3—C2	46.5 (3)	C6—Fe1—C9—C8	80.65 (12)
C10—Fe1—C3—C2	−168.23 (18)	C10—Fe1—C9—C8	119.43 (16)
C1—Fe1—C3—C2	−37.90 (14)	C3—Fe1—C9—C8	−79.82 (14)
C4—Fe1—C3—C2	−119.3 (2)	C2—Fe1—C9—C8	−47.1 (2)
C5—Fe1—C3—C2	−81.58 (15)	C1—Fe1—C9—C8	167.7 (3)
C7—Fe1—C3—C2	76.51 (16)	C4—Fe1—C9—C8	−122.32 (13)
C8—Fe1—C3—C2	117.25 (14)	C5—Fe1—C9—C8	−162.70 (13)
C9—Fe1—C3—C4	−81.75 (16)	C7—Fe1—C9—C8	37.01 (12)
C6—Fe1—C3—C4	165.8 (2)	C6—Fe1—C9—C10	−38.79 (11)
C10—Fe1—C3—C4	−48.9 (3)	C3—Fe1—C9—C10	160.74 (11)
C2—Fe1—C3—C4	119.3 (2)	C2—Fe1—C9—C10	−166.50 (17)
C1—Fe1—C3—C4	81.39 (16)	C1—Fe1—C9—C10	48.3 (3)
C5—Fe1—C3—C4	37.71 (14)	C4—Fe1—C9—C10	118.25 (12)
C7—Fe1—C3—C4	−164.21 (14)	C5—Fe1—C9—C10	77.86 (14)
C8—Fe1—C3—C4	−123.46 (15)	C7—Fe1—C9—C10	−82.42 (12)
C2—C3—C4—C5	−0.3 (2)	C8—Fe1—C9—C10	−119.43 (16)
Fe1—C3—C4—C5	−59.83 (15)	C8—C9—C10—C6	−0.8 (2)
C2—C3—C4—Fe1	59.54 (15)	Fe1—C9—C10—C6	59.25 (12)
C9—Fe1—C4—C5	−125.37 (13)	C8—C9—C10—C11	−176.81 (17)
C6—Fe1—C4—C5	−48.5 (3)	Fe1—C9—C10—C11	−116.81 (17)
C10—Fe1—C4—C5	−82.20 (15)	C8—C9—C10—Fe1	−60.00 (13)
C3—Fe1—C4—C5	118.7 (2)	C7—C6—C10—C9	0.5 (2)
C2—Fe1—C4—C5	81.21 (14)	Fe1—C6—C10—C9	−59.14 (12)
C1—Fe1—C4—C5	37.49 (13)	C7—C6—C10—C11	176.06 (18)
C7—Fe1—C4—C5	160.6 (2)	Fe1—C6—C10—C11	116.4 (2)
C8—Fe1—C4—C5	−166.69 (13)	C7—C6—C10—Fe1	59.63 (13)
C9—Fe1—C4—C3	115.94 (15)	C6—Fe1—C10—C9	117.91 (15)
C6—Fe1—C4—C3	−167.2 (2)	C3—Fe1—C10—C9	−45.2 (2)
C10—Fe1—C4—C3	159.12 (13)	C2—Fe1—C10—C9	160.7 (2)
C2—Fe1—C4—C3	−37.47 (14)	C1—Fe1—C10—C9	−164.44 (12)
C1—Fe1—C4—C3	−81.20 (15)	C4—Fe1—C10—C9	−80.36 (14)
C5—Fe1—C4—C3	−118.7 (2)	C5—Fe1—C10—C9	−122.90 (13)
C7—Fe1—C4—C3	41.9 (3)	C7—Fe1—C10—C9	80.68 (12)
C8—Fe1—C4—C3	74.63 (17)	C8—Fe1—C10—C9	37.27 (12)
C3—C4—C5—C1	0.2 (2)	C9—Fe1—C10—C6	−117.91 (15)
Fe1—C4—C5—C1	−59.39 (15)	C3—Fe1—C10—C6	−163.1 (2)
C3—C4—C5—Fe1	59.57 (15)	C2—Fe1—C10—C6	42.8 (3)
C2—C1—C5—C4	0.0 (2)	C1—Fe1—C10—C6	77.64 (14)
Fe1—C1—C5—C4	59.44 (15)	C4—Fe1—C10—C6	161.73 (12)
C2—C1—C5—Fe1	−59.44 (15)	C5—Fe1—C10—C6	119.19 (12)
C9—Fe1—C5—C4	73.43 (16)	C7—Fe1—C10—C6	−37.23 (11)
C6—Fe1—C5—C4	160.41 (13)	C8—Fe1—C10—C6	−80.64 (12)
C10—Fe1—C5—C4	116.66 (14)	C9—Fe1—C10—C11	115.73 (19)
C3—Fe1—C5—C4	−38.14 (14)	C6—Fe1—C10—C11	−126.36 (19)
C2—Fe1—C5—C4	−81.87 (15)	C3—Fe1—C10—C11	70.5 (3)
C1—Fe1—C5—C4	−119.4 (2)	C2—Fe1—C10—C11	−83.6 (3)
C7—Fe1—C5—C4	−162.5 (2)	C1—Fe1—C10—C11	−48.71 (18)
C8—Fe1—C5—C4	36.5 (3)	C4—Fe1—C10—C11	35.37 (18)
C9—Fe1—C5—C1	−167.19 (13)	C5—Fe1—C10—C11	−7.17 (17)
C6—Fe1—C5—C1	−80.21 (16)	C7—Fe1—C10—C11	−163.58 (17)
C10—Fe1—C5—C1	−123.96 (14)	C8—Fe1—C10—C11	153.00 (16)
C3—Fe1—C5—C1	81.24 (15)	N2—N1—C11—C10	2.6 (3)
C2—Fe1—C5—C1	37.51 (14)	N2—N1—C11—C12	−176.20 (16)
C4—Fe1—C5—C1	119.4 (2)	C9—C10—C11—N1	−171.86 (19)
C7—Fe1—C5—C1	−43.2 (3)	C6—C10—C11—N1	13.2 (3)
C8—Fe1—C5—C1	155.9 (2)	Fe1—C10—C11—N1	103.5 (2)
C9—Fe1—C6—C7	−80.80 (12)	C9—C10—C11—C12	6.9 (3)
C10—Fe1—C6—C7	−119.61 (16)	C6—C10—C11—C12	−168.03 (18)
C3—Fe1—C6—C7	40.1 (3)	Fe1—C10—C11—C12	−77.7 (2)
C2—Fe1—C6—C7	74.84 (14)	N4—N3—C13—N2	1.5 (3)
C1—Fe1—C6—C7	117.07 (12)	N4—N3—C13—C14	−179.9 (2)
C4—Fe1—C6—C7	−165.2 (2)	C15—N2—C13—N3	−1.8 (2)
C5—Fe1—C6—C7	159.45 (12)	N1—N2—C13—N3	−171.75 (18)
C8—Fe1—C6—C7	−37.23 (12)	C15—N2—C13—C14	179.5 (2)
C9—Fe1—C6—C10	38.81 (11)	N1—N2—C13—C14	9.5 (3)
C3—Fe1—C6—C10	159.7 (2)	N3—N4—C15—N2	−0.6 (3)
C2—Fe1—C6—C10	−165.55 (11)	N3—N4—C15—C16	179.7 (2)
C1—Fe1—C6—C10	−123.32 (11)	C13—N2—C15—N4	1.5 (2)
C4—Fe1—C6—C10	−45.6 (3)	N1—N2—C15—N4	171.25 (18)
C5—Fe1—C6—C10	−80.93 (13)	C13—N2—C15—C16	−178.9 (2)
C7—Fe1—C6—C10	119.61 (16)	N1—N2—C15—C16	−9.1 (3)

### Hydrogen-bond geometry (Å, °)

**Table:**

*D*—H···*A*	*D*—H	H···*A*	*D*···*A*	*D*—H···*A*
C6—H6···Cg1	0.98	2.52	3.255 (3)	132.
C5—H5···Cg3^i^	0.98	2.87	3.703 (5)	144.

Symmetry codes:  (i) *x*, −*y*−1/2, *z*−1/2.
